# Gigantic Multiple Odontomas of Jaws in a Pediatric Patient: A Rare Case

**DOI:** 10.7759/cureus.63274

**Published:** 2024-06-27

**Authors:** Sheetal Badnaware, Vinay Kumar Srivastava

**Affiliations:** 1 Pedodontics and Preventive Dentistry, Faculty of Dental Sciences, Institute of Medical Sciences, Banaras Hindu University, Varanasi, IND

**Keywords:** gigantic odontomas, compound odontoma, delayed tooth eruption, odontomatosis, multiple odontomas

## Abstract

Odontomas are mixed epithelial and mesenchymal tumor-like malformations (hamartoma) composed of dental hard and soft tissue, causing delayed teeth eruption. Multiple case reports have been published in the literature describing solitary, localized odontoma features in pediatric patients along with their treatment planning. This report presents clinical and radiographic features of gigantic multiple odontomas involving both jaws in a five-year-old boy.

## Introduction

Odontomas are well-known among dental practitioners. These odontomas are mixed odontogenic tumors in which both the epithelial and mesenchymal components are functionally differentiated to the extent that both enamel and dentin have developed. Odontomas are thought to be hamartomatous rather than tumorous in nature [1]. Multiple odontomas, odontomatosis, or odontoma syndrome are characterized by odontomas involving one or more quadrants of the jaw [2]. Radiographic diagnosis of single or more tooth-like structure malformations (odontomas) is not difficult. However, the amorphous presence of a calcified tooth-like structure involving all quadrants of the jaw complicates the diagnosis. There are two basic types of odontomas: compound and complex. The specific location within the jaw can vary, but odontomas are generally found where dental tissues develop in the anterior and posterior regions of both jaws. Multiple odontomas of the mixed variety are usually seen in the maxillary or mandibular region and both, and numerous case reports are in the literature [2]. The present article describes a case of multiple odontoma involving both jaws in a five-year-old boy.

## Case presentation

**Figure 1:**
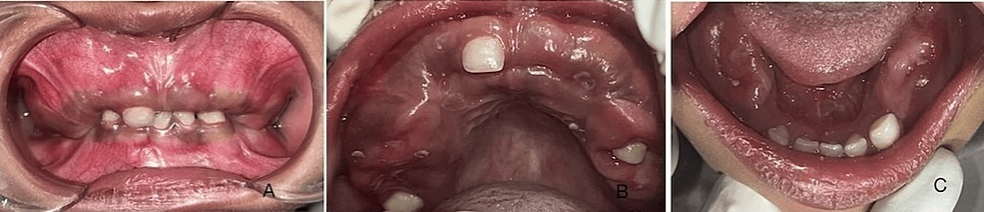
Intraoral view showing multiple missing deciduous teeth. A) frontal, B) Maxilla, and C) Mandible

A five-year-old boy presented with a chief complaint of multiple unerupted teeth in both jaws, noted since six to seven months of age. The child was well-built and in good health. Medical history was non-contributory. Upon extraoral examination, no swelling or facial asymmetry was observed. An intraoral examination found fully erupted only deciduous right maxillary central incisor, deciduous right and left mandibular central and lateral incisors, and deciduous left mandibular canine (Figure 1). Two cusp tips were seen clinically and radiographically which is difficult to diagnose. The remaining deciduous teeth were found missing. Family history was non-contributory. Clinical examination showed maxillary and mandibular areas with hard bony expansion of jaws.

**Figure 2:**
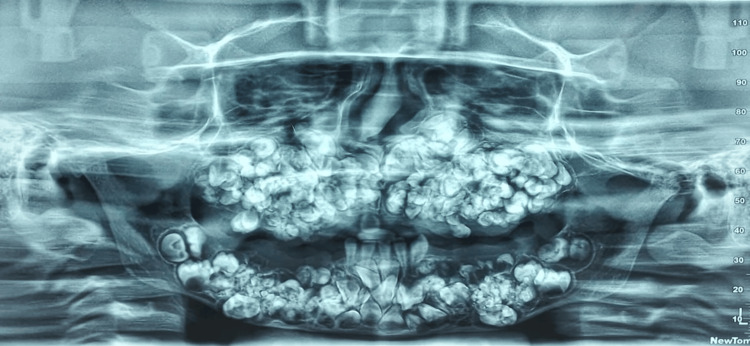
Orthopantomograph shows the presence of multiple calcified tooth-like structures.

An orthopantographic examination revealed the presence of multiple calcified tooth-like structures in all quadrants of the jaws with more in the maxilla than the mandible (Figure 2).

**Figure 3:**
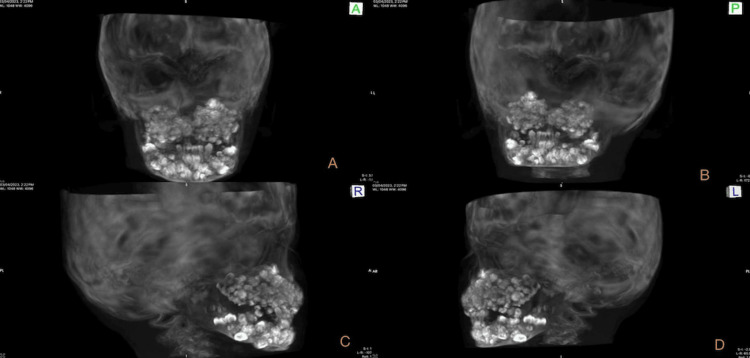
3D scan of cone-beam computed tomography (CBCT) in different views A) Anterior view, B) Posterior view, C) Right lateral view, and D) Left lateral view showing multiple tooth-like structures.

The density of the calcified structure was similar to the tooth-like structure suggesting compound-composite odontomas. Some permanent teeth on the radiograph were found to be displaced or unerupted, but identifying them was difficult on the radiograph due to the superimposition of multiple calcified tooth-like structures over each other. A 3D view of cone-beam computed tomography (CBCT) also showed the presence of numerous calcified tooth-like structures involving both jaws (Figure 3). Multiple unerupted permanent teeth and impacted deciduous teeth were also observed (Figure 3).

Routine blood examination like complete blood count and biochemical assessments like serum calcium, serum phosphorous, serum alkaline phosphatase, serum glutamic oxaloacetic transaminase, thyroxine (T3), triiodothyronine (T4), and thyroid-stimulating hormone (TSH) were within normal ranges. The referrals to a pediatrician and orthopedic surgeon ruled out the possibility of Gardner syndrome or any bony pathology. Based on the clinical and radiographical presentation, a final diagnosis of multiple odontoma or odontomatosis was made. After consulting with an oral surgeon, the parents preferred not to undergo treatment at this time. Only symptomatic and preventive treatments such as oral hygiene instructions, were given to the patient and parents. The child’s masticatory efficiency is compromised due to the extensive size of the lesion; hence, a soft diet has been advised. The parents were advised to schedule regular check-ups. Here, we presented a rare case of multiple unerupted tooth-like radiopacities affecting both jaws in pediatric patients.

## Discussion

Odontomas are an important clinical entity among odontogenic tumors. In the present case, multiple tooth-like radiopacities were observed in both jaws diagnosed as odontomatosis or multiple odontoma. The presented case is very unusual as very few reports of similar cases have been identified in the literature. Bader (1967) and Mani (1974) have been given identical case reports in the literature involving all four quadrants. Based on both radiographical and clinical appearance, Mani suggested different terminology for such gigantic lesions like multiple odontomas or odontoma syndrome in the literature [3,4]. Differential diagnoses must include ameloblastic fibro odontoma, odontoamleoblastoma, or ameloblastic fibroma. The exact etiology of odontoma remains unknown, although trauma, infection, or genetic or hereditary syndromes such as Gardner’s syndrome and Hermann’s syndrome have been suggested [5]. Odontomas are often asymptomatic and routinely diagnosed via radiograph, showing jaw expansion, facial swelling, and delayed eruption of permanent teeth [5]. Surgery is employed to effectively remove multiple odontomas while minimizing the risk of recurrence. A two-phase surgical intervention has been described in the literature for giant odontomas, with a higher risk of pathological fracture and injury to essential anatomical structures [2]. To date, there is no general agreement on the treatment of impacted permanent teeth in these lesions. Treatment options include extraction, surgical repositioning, and clinical and radiographic monitoring of the teeth. This case report is notable because very few cases of such gigantic odontomas in pediatric patients have been published in the literature. Early detection of such kind of lesion is crucial, as this lesion can predispose to cystic changes. Lesions of this gigantic size with this clinical and radiographic presentation have not been widely reported in pediatric patients, and impacted teeth could be multiple.

## Conclusions

Early diagnosis and radiographic examination increase the chances of preserving impacted permanent teeth. The literature suggests that radiographic examination should be done for any pediatric patients who present with chief complaints of delayed eruption of teeth or impacted teeth. Multiple odontomas are not part of everyday clinical practice, making it extremely important for pediatric dentists to recognize such a rare and unusual presentation.
